# Design of uncertain displacement controlled velocity control system for hydraulic actuator

**DOI:** 10.1016/j.heliyon.2024.e26223

**Published:** 2024-02-14

**Authors:** Jabbar H. Mohmmed, Ahmed K. Hassan, Muslim Ali, Samer A. Kokz, Muhanad Hamed Mosa, A.K. Kareem, Ahmed A. Zainulabdeen, Z.C. Feng

**Affiliations:** a Materials Engineering Department, University of Technology- Iraq, Baghdad, Iraq; b Prosthetics and Orthotics Engineering Department, College of Engineering, University of Kerbala, Iraq; c College of Engineering, University of Warith Al-Anbiyaa, Karbala, Iraq; d Mechanical Engineering Department, College of Engineering, University of AlQadisiyah, Iraq; e Biomedical Engineering Department, Al-Mustaqbal University College, 51001, Hillah, Iraq; f Mechanical and Aerospace Engineering, University of Missouri, USA

**Keywords:** Controller, Hydraulic, Actuator, Pump, Disturbance transfer function

## Abstract

Displacement-controlled systems have high efficiency and are widely used in industry. Accurate control of the actuator motion in hydraulic systems is usually a necessity in industrial applications such as the motion of control surfaces in fixed-wing airplanes for flight control as well as the aircraft brake systems. To address this need, the current study was conducted with the goal of developing a high-fidelity model to achieve precise control. This work focused on modeling a hydrostatic transmission that is used for controlling a linear actuator velocity. The flow entering the actuator was changed using a variable displacement pump. The study included examining the stability and performance of the open-loop system. Additionally, the study involved the design of the proportional-integral-derivative ***PID*** and ***H***∞ controllers, followed by the analysis of the stability and performance of the closed-loop system with both controllers. Furthermore, the multiplicative uncertainty is taken into account and the robustness of the system is verified using controllers ***PID*** and ***H***∞. In the current study,Uncertain parameters such as actuator efficiency, pump speed, and viscous friction coefficient were considered and allowed for a ±5% deviation from their stated values. Taking uncertainty into account ensures that the system performs properly even in case where the design parameters vary within the specified range. The system response is compared for the cases of open-loop system, closed-loop system with ***PID*** controller, and closed-loop system with ***H***∞ controller. The results demonstrated that the open-loop system remains stable for real-world applications but shows insufficient performance in terms of input tracking and disturbance rejection. The introduction of the PID controller significantly enhanced the system's response to a reference input; however, its disturbance rejection capabilities in terms of overshoot and settling time were still unsatisfactory. The system equipped with the PID controller failed to meet the robustness requirements. Conversely, the utilization of $H_{\infty }$ controllers yielded superior responses and fulfilled the robustness criteria.

## Nomenclature

A: = Maximum Low Frequency Error
$A_{A}$: = Actuator Cross Sectional Area
c: = Viscous Damping Coefficient
D: = Disturbance Transfer Function
E: = Error Signal
F: = Disturbance Force
$F_{o}$: = Gravitational Force
G: = Plant Transfer Function
K: = H_∞_ Controller Transfer Function
$K_{P}$: = Proportional Controller Gain
$K_{i}$: = integral Controller Gain
$K_{d}$: = Derivative Controller Gain
k: = Leakage Coefficient
$l_{1}$: = Multiplicative Uncertainty
M: = Maximum High Frequency Error
***m***: *=* Load Mass
***P***: = Pressure
P: = Generalized Plant
Q: = Volume Flow Rate
S: = Sensitivity
$V_{o}$: = Volume of the Actuator
$V_{R}$: = Reference Input
$v_{a}$: = Cylinder Velocity
w: = Exogenous Input
$w_{1}$: = Uncertainty Weight Transfer Function
x: = Actuator Displacement
z: = Exogenous Output
β: = Fluid Bulk Modulus
$\eta_{a}$: = Efficiency of the Actuator
$\omega_{b}$: = Bandwidth Frequency
$w_{p}$: = Performance weight
$w_{u}$: = Controller Effort Weight
τ: = Time Constant
$\varphi_{1}$: = Nondimensional group
$\varphi_{2}$: = Nondimensional group
${\left(\right)}_{A}$: = Quantity Measured at Port A of the actuator
${\left(\right)}_{B}$: = Quantity Measured at Port B of the actuator
${\left(\right)}_{p}$: = Pump
${\left(\right)}_{r}$: = Reference Conditions
(ˆ): = Dimensionless Quantity

## 1 Introduction

Hydrostatic transmission is widely used in industrial applications such as off-high way vehicles, airplanes, etc. In all those applications, a precise control over the motion of the hydraulic actuator is required. Hydrostatic Transmission used to transmit power using pressurized fluid. A hydrostatic transmission typically includes a pump that converts mechanical power in the place where it is generated into fluid power and an actuator that converts fluid power into mechanical power in the place of use. In addition to the pump and actuator, a hydrostatic transmission may include some accessories such as hoses, valves, etc. Hydrostatic transmissions are usually used for a variety of control objectives, including position control, velocity control, and force control. In most cases, a precise control is required as in the control of the motion of control surfaces of the fixed-wings airplanes. These objectives are accomplished by modifying the flow entering the hydraulic actuator. Flow is controlled by using either valve-controlled systems or pump-controlled systems. Although less complicated and cheaper, valve control systems are less efficient than pump control systems due to power losses across the throttle valves. Displacement controlled systems are another type of pump controlled systems. They are efficient and have good control characteristics [1].

Numerous studies have been made to design hydrostatic transmissions using valve-controlled systems. For velocity control of a linear hydraulic actuator, Zhang [2] showed that the feedforward plus PID (FPID) controller R-square is lower than that of the feedforward loop and the PID controller. In addition, it was revealed that the system stability and performance were improved by using the FPID controller. Lin [3] demonstrated that the using of an electrohydraulic proportional control system for velocity control has good performance characteristics and can track inputs accurately within a half second. Maneetham [4] and Hassan et al. [5,6] showed that better results in controlling the velocity and position of the hydraulic servo system and automotive engine can be achieved using a hybrid controller (combination of PID or PI control and fuzzy control) than using them both separately. Pan [7,8] performed studies on what is known inertance tube switched systems. He found that these systems experience significant levels of noise and necessitate the use of extremely rapid switching valves. Humaidi and Oleiwi [9] utilize PID controller for velocity control of a hydraulic motor system with a swashplate DC-controlled pump. Ali [10] observed that the inlet throttling system, in the system in which the throttling valve is placed upstream from the pump, may suffer from cavitation effects due to the starving of the pump inlet. He showed that the efficiency of the inlet metered pump may exceed 80% depending on the opening of the throttle valve and the pump speed. Aranovskiy et al. [11] presented an experimentally validated model of a nonlinear model was introduced for the purpose of controlling an actuator position using valve control method. Feedforward control was used to improve the system response. An adaptive sliding mode control strategy was utilized in Ref. [12] to control the motion of an electrohydraulic actuator using. The system stability was assessed using Lyapunov theory and the simulation was performed in Matlab/Simulink®.

Waheed et al. [13,14] proved that using a modified method of adaptive controlling, which consist of two parts (inverse neural control and adaptive correction factor or adaptive gain factor), can successfully control the DC motor velocity. Çalişkan et al. [15] conducted a study on the implementation of a variable speed drive for position control in a hydraulic system. Kalman filter to reduce the noise in the feedback signal was used. They notice more energy efficiency in the case of a valveless hydraulic circuit as compared to the losses associated with the valve-controlled system. Mahdi and Lutfy [16] demonstrated that employing an extended wavelet functional link neural network (EWFLNN) for controlling the displacement response of a servo-hydraulic system yields superior control precision compared to other controllers, such as PID controller, artificial neural network (ANN) controller, wavelet neural network (WNN) controller, and the original wavelet functional link neural network (WFLNN) controller. A comprehensive study of the efficiency hydrostatic transmission that uses a variable displacement pump and a rotor hydraulic actuator was performed by Manring [17]. The study presented efficiency maps for the pump, motor, and the overall hydrostatic transmission. Those maps can be used as a reference to understand the behavior of those machines. The findings indicated that the efficiency of a displacement-controlled hydrostatic transmission may exceed 87%. Furthermore, it was shown that the machine efficiency may remain unaffected by either speed or torque, contingent upon the specific operating conditions. In Ref. [18], the flow control methods for hydraulic system were reviewed. It was stated that displacement controlled systems have the highest efficiency compared to other flow control strategies. Displacement controlled systems are widely used due to their high efficiency in addition to the minimized heat exchanger needs [19].

In this research, a high fidelity model was created for a hydrostatic transmission employed for controlling the velocity of a linear actuator using a variable displacement pump. The open-loop system's stability and performance were investigated. In order to get better the system performance, PID and $H_{\infty }$ controllers were designed. The closed-loop system's stability and performance , incorporating the PID and $H_{\infty }$ controllers, were analyzed. Moreover, a comparative analysis of the system responses for the three cases was presented. In order to ensure that the system meets the performance requirements when changes in the systems parameters exist, the parametric uncertainty associated with the system was considered and the robustness of the system with both the PID and the $H_{\infty }$ controllers was assessed. Excellent performance was achieved in terms of the response speed, percent overshoot, disturbance rejection, and robustness compared to the existing designs.

## 2 System description

**Fig. 1:**
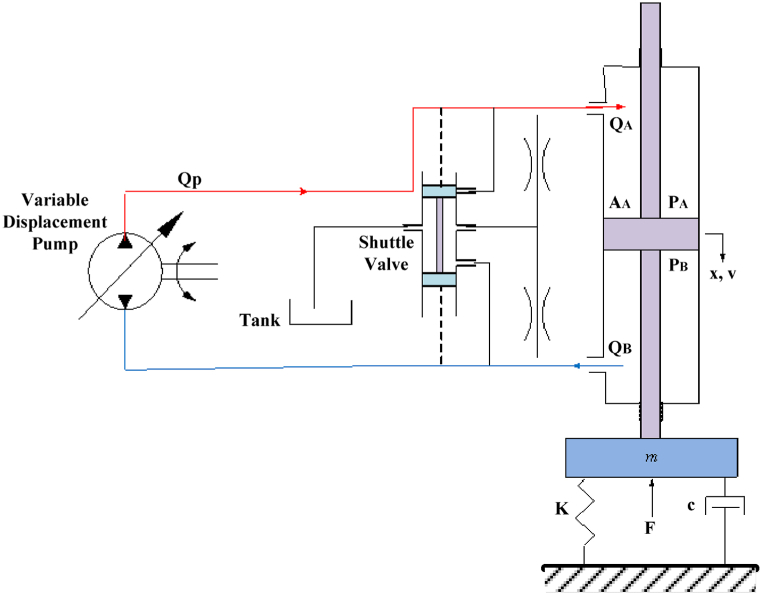
Displacement controlled velocity control system.

Fig. 1 shows the displacement-controlled hydrostatic transmission used for speed control purposes. It consists of a variable displacement pump, a linear actuator, and accessories (hoses, safety valves, etc.). As shown in Fig. 1, a single system with mass-spring-damper and a disturbance force was used to represent the load that the actuator moves, where m,k, and c are the mass of the load, the stiffness of the spring, and the viscous drag coefficient, respectively. A double-bar linear actuator is used with equal areas on both sides so that the actuator action is symmetrical. The control of the actuator's velocity involves the adjustment of the flow entering side A of the actuator. Whereas the flow is adjusted by altering the displacement of the pump by changing the dimensionless switchboard angle. $\left(\hat{\alpha }=\frac{\alpha }{\alpha_{\max}}\right)$ which ranges from −1 to 1 where α is the instantaneous swashplate angle and $\alpha_{\max}$ is maximum swashplate angle. A full description of variable displacement pumps for the swashplate mechanism is found in Ref. [19]. For a positive switchboard angle, the fluid flows to side, which moves the load down and flows back from side B to the actuator. For a negative switchboard angle, the opposite happens. The shuttle valve in Fig. 1 connects the low-pressure side of the hydraulic circuit to the tank. So that it maintains the low-pressure side at zero pressure and helps to avoid fluid cavitation which may be caused by vacuum drawing by the pump. In addition, it allows the use of a low-pressure radiator (not displayed in Fig. 1) for return flow cooling [20]. The dashed lines in Fig. 1 indicate that the pressure signals are used to cause the shuttle valve to move in the direction specified by the flow direction. This is done by pressure signals indicated by the dashed lines. The schematic in the leakage coefficient k represents the leakage that exists on both sides of the system.

## 3 System modeling

The pressure on the side A of the actuator is assumed to be equal to the discharge pressure of the pump $\left(P_{A}=P_{d}\right)$. The system is modeled as if there is a single volume extending from the pump discharge to the piston surface. The behavior of the system is governed by the actuator equation of motion and the equation of the pressure rise rate. The equation of motion can be described as follows:

(1) $$m\ddot{x}+c\dot{x}+Kx-mg=\eta_{a}P_{A}A_{A}-F_{0}-F\text{,}$$

Where *m*, *c*, and *K* are the load mass, viscous friction coefficient, and spring stiffness respectively, *P*_*A*_ and *A*_*A*_ are the pressure and area of side A of the actuator, *F* is the external disturbance, and *F*_*0*_ is the spring pretension. In steady state operation, $x=\dot{x}=\ddot{x}=F=P_{A}=0$. These yields $F_{o}=mg$ which will be considered for all cases. In addition, the analysis of velocity control does not incorporate the load spring [16], then Eq. (1) becomes,

(2) $$m{\dot{v}}_{a}+cv_{a}=\eta_{a}P_{A}A_{A}-F\text{,}$$

In Eq. (2), the derivative of the actuator displacement, $\dot{x}$, was replaced by the actuator velocity, $v_{a}$ , the cylinder velocity. Equation (3) represents the pressure rise rate equation:

(3) $${\dot{P}}_{A}=\frac{\beta }{V_{o}+A_{A}x}\left(Q_{P}-kP_{A}-A_{A}v_{a}\right)$$

where the flow rate of the pump, denoted as $Q_{P}$, which is defined $Q_{p}=D_{p}\omega \hat{\alpha }$ where $D_{p}$ represents the pump displacement and $\hat{\alpha }$ is the dimensionless swashplate angle of the pump $\left(\hat{\alpha }=\frac{\alpha }{\alpha_{\max}}\right)$.

The leakage losses within the system are represented by the leakage coefficient, denoted as k. In this analysis, the leakage coefficient is assumed to remain constant and its value is taken as one-tenth of the ratio of the maximum volumetric flow rate to the maximum pressure, $k=\frac{Q_{\max}}{10P_{\max}}$ [21].

A linearized form of Eq. (3) will be used in the present work, which is achieved using the following nominal conditions: $x_{o}=v_{a}=Q_{p_{o}}=P_{A_{o}}=0$. That yields,

(4) $${\dot{P}}_{A}=\frac{\beta }{V_{o}}\left(Q_{P}-kP_{A}-A_{A}v_{a}\right)$$

Substituting for the flow rate, $Q_{p}$, Eq. (4) can be written as:

(5) $${\dot{P}}_{A}=\frac{\beta }{V_{o}}\left(D_{p}\omega \hat{\alpha }-kP_{A}-A_{A}v_{a}\right)$$

For model generalization purposes, Equations (2), (5) were written in a dimensionless form using the following reference condition,

$$P_{A}={\hat{P}}_{A}P_{A_{r}},v_{a}={\hat{v}}_{a}v_{a_{r}}\;and\;t=\hat{t}\tau$$

The dimensionless form of Eq. (5) may be expressed as:

(6) $$\frac{d\left({\hat{P}}_{A}P_{A_{r}}\right)}{d\left(\hat{t}\tau \right)}=\frac{\beta }{V_{o}}\left(D_{p}\omega \hat{\alpha }-k{\hat{P}}_{A}P_{A_{r}}-A_{A}v_{a_{r}}{\hat{v}}_{a}\right)$$

Equation (6) can be simplified by multiplying by $\frac{\tau }{P_{A_{r}}}$ which gives:

(7) $${\hat{\dot{P}}}_{A}=\frac{\tau \beta }{V_{o}P_{A_{r}}}\omega D_{p}\hat{\alpha }-\frac{\tau \beta }{V_{o}}k{\hat{P}}_{A}-\frac{\tau \beta v_{a_{r}}}{V_{o}P_{A_{r}}}A_{A}{\hat{v}}_{a}$$

If the time constant is defined as τ $=\frac{V_{o}}{\beta k}$, then, the dimensionless pressure rise equation may be expressed as the following

(8) $${\hat{\dot{P}}}_{A}+{\hat{P}}_{A}=\varphi_{1}\hat{\alpha }-\varphi_{2}{\hat{v}}_{a}$$

where the dimensionless quantities $\varphi_{1}$ and $\varphi_{2}$ in Eq. (8) are given as,

$$\varphi_{1}=\frac{\omega D_{p}}{kP_{A_{r}}}$$

$$\varphi_{2}=\frac{v_{a_{r}}A_{A}}{kP_{A_{r}}}$$

In a similar manner, Eq. (2) may be expressed in a dimensionless form using the above-mentioned reference conditions,

(9) $$m\frac{d\left({\hat{v}}_{a}v_{a_{r}}\right)}{d\left(\hat{t}\tau \right)}+c{\hat{v}}_{a}v_{a_{r}}=\eta_{a_{f}}P_{A}A_{A}-F$$

The final form of the dimensionless equation of motion is determined by dividing Eq. (9) by $P_{A_{r}}A_{A}$ which yields,

(10) $$\hat{m}{\dot{\hat{v}}}_{a}+\hat{c}{\hat{v}}_{a}=\eta_{a}{\hat{P}}_{A}-\hat{F}$$

Where

$$\left\{\begin{array}{c} \hat{m}=\frac{mv_{a_{r}}}{P_{A_{r}}A_{A}\tau }\\ \hat{c}=\frac{cv_{a_{r}}}{P_{A_{r}}A_{A}}\\ {\hat{A}}_{A}=1\\ \hat{F}=\frac{F}{P_{A_{r}}A_{A}} \end{array}\right\}$$

Table 1 list the values of the quantities used in the simulation.

**Table 1:** Values of the simulation parameter.

Symbol	Dimensional Value	Dimensionless Value
$A_{A}$	3.5E-4 $m^{2}$	1
c	3000 N.s/m	0.1
$D_{p}$	10 $cm^{3}/rev$	–
F	12,000 N	0.8
k	1E-12 $m^{4}s/kg$	–
m	400 kg	0.26
$P_{A_{r}}$	30E+6 $P_{a}$	1
$v_{R}$	0.5 m/s	0.5
β	2E9 $P_{a}$	–
τ	0.25 s	–
$\varphi_{1}$	–	10
$\varphi_{2}$	–	8.33
ω	1800 rpm	–

## 4 Control design and performance analysis

**System Stability.** Equations (8), (10) may be described in state-space form as follows,

$$\dot{x}=Ax+Bu\;\text{and}\;y=Cx+Du$$

where $x={\left[{\hat{P}}_{A}{\hat{v}}_{a}\right]}^{T},u={\left[\hat{\alpha }\hat{F}\right]}^{T}and\;y={\hat{v}}_{a}$. In addition, the matrices A,B,C,andD defined based on Equations. (8 and 10),

$$A=\left[\begin{array}{cc} -1 & -\varphi_{2}\\ \frac{\eta_{a}}{\hat{m}} & -\frac{\hat{c}}{\hat{m}} \end{array}\right]$$

$$B=\left[\begin{array}{cc} \varphi_{1} & 0\\ 0 & -\frac{1}{\hat{m}} \end{array}\right]$$

C=[01]and

D=0

The characteristic equation of the system may be written as:

$$\det\left(sI-A\right)=s^{2}+\left(\frac{\hat{c}}{\hat{m}}+1\right)s+\left(\frac{\hat{c}+\eta_{a}\varphi_{2}}{\hat{m}}\right)=0$$

Let,

(11) $$\left\{\begin{array}{c} a_{o}=1\\ a_{1}=\frac{\hat{c}}{\hat{m}}+1\\ a_{2}=\frac{\hat{c}+{\hat{A}}_{A}^{2}\eta_{a}\varphi_{2}}{\hat{m}} \end{array}\right\}$$

The Routh-Hurwitz stability criterion was employed to assess the stability of the system. According to this criterion, the coefficients in Equation (11) must be positive for the system to be considered stable., i. e,

$$a_{0,1,2}>0$$

Since all definitions in Eq. (11) are positive in the real world applications, then, this system will always remain stable for any realistic choice of the design parameters.

## 5 System performance

To evaluate the system's performance, the initial step involved analyzing the response of the open-loop system. Subsequently, both PID and $H_{\infty }\text{,}$ controllers were designed to further investigate the system's behavior. The responses of the system with both controllers were carefully examined and compared to each other, as well as to the open-loop system response. Additionally, the multiplicative parametric uncertainty of the system was analyzed, and the robustness of the system was evaluated.

### 5.1 Open-loop system

**Fig. 2:**
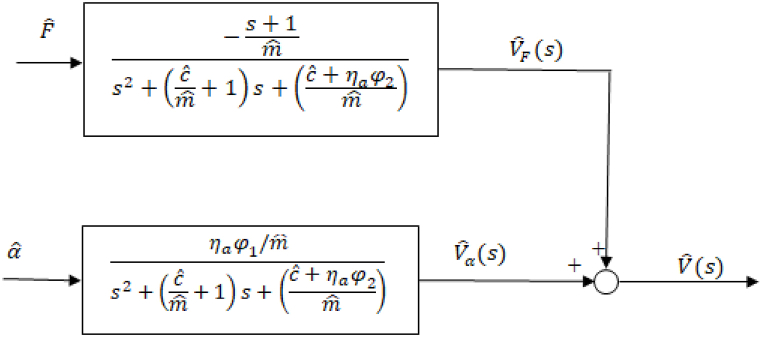
Open-loop system block diagram.

Fig. 2 illustrates the open-loop system block diagram in which D(s) is a disturbance force. The transfer function of the system that relates the output velocity, *v*_*α*_, to the dimensionless pump swashplate angle, $\hat{\alpha }$, is expressed in Eq. (12):

(12) $$G\left(s\right)=\frac{{\hat{V}}_{\alpha }}{\hat{\alpha }}=\frac{\eta_{a}\varphi_{1}/\hat{m}}{s^{2}+\left(\frac{\hat{c}}{\hat{m}}+1\right)s+\left(\frac{\hat{c}+\eta_{a}\varphi_{2}}{\hat{m}}\right)}$$

Furthermore, the transfer function that describes the relationship between the disturbance force and the output velocity that is resulted from that disturbance, *v*_*F*_, is given in Eq. (13):

(13) $$D\left(s\right)=\frac{{\hat{V}}_{F}\left(s\right)}{\hat{F}\left(s\right)}=\frac{-\frac{s+1}{\hat{m}}}{s^{2}+\left(\frac{\hat{c}}{\hat{m}}+1\right)s+\left(\frac{\hat{c}+\eta_{a}\varphi_{2}}{\hat{m}}\right)}$$

### 5.2 Closed-loop system

**Fig. 3:**
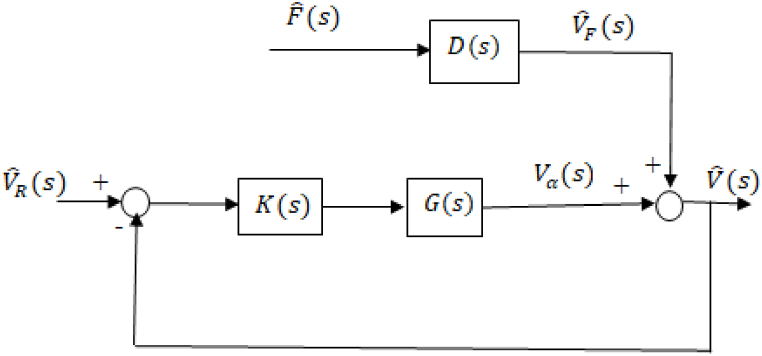
Closed-loop system block diagram.

The closed-loop system block diagram is shown in Fig. 3 with K(s) being the controller block. PID controller was used first. Auto-tuning with MATLAB/SIMULINK® was used for the purpose of determining the controller gains.

**Fig. 4:**
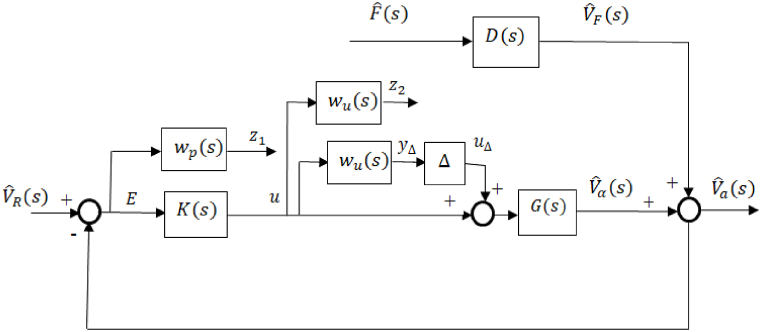
The block diagram of the closed-loop system with the weights.

**Design Process of the**$H_{\infty }$**Controller**: It was observed that the system's response exhibited significant overshoot and oscillation when controlled by the PID controller. To enhance the system's performance, an $H_{\infty }$ controller was designed. The design process of the $H_{\infty }$ controller is outlined below, along with a depiction of the closed-loop system block diagram in Figure (4), where $w_{p}\left(s\right)$ represents the performance weight, and $w_{u}\left(s\right)$ represents the control effort weight.

The performance weight, $w_{p}\left(s\right)$, is defined as the inverse of the upper bound of the sensitivity function magnitude, S(s) which is given as [22],

(14) $$w_{p}\left(s\right)=\frac{\frac{s}{M}+{\hat{\omega }}_{b}}{s+A{\hat{\omega }}_{b}}$$

In Equation (14), the variables ${\hat{\omega }}_{b}$, M, and A represent the dimensionless bandwidth frequency, high-frequency error, and low-frequency error, respectively. It is important to note that the control design aims to minimize the low-frequency error to achieve accurate input tracking while maintaining satisfactory disturbance rejection. The literature provides a range of values for M. For example, Carpenter [18] used a value of 1.5, while Fales [23] employed a value of 6. Carpenter [22] also suggested a reasonable value of 0.01 for the low-frequency error, A. In this study, the specific parameter values selected are as follows: A = 0.01, M = 2, and ${\hat{\omega }}_{b}$ = 0.3. Furthermore, a reasonable choice for the controller effort weight in dimensionless systems is 1. The sensitivity function, denoted as S, represents the transfer function that links the reference signal to the error signal and can be mathematically expressed as [24]:

(15) $$S\left(s\right)=\frac{E\left(s\right)}{{\hat{V}}_{R}\left(s\right)}=\frac{1}{1+G\left(s\right)K\left(s\right)}$$

The symbol S, in Eq. (15), represents the sensitivity function. Furthermore, the transfer function relating the disturbance signal to the error signal given in Eq. (16):

(16) $$\frac{E\left(s\right)}{\hat{F}\left(s\right)}=-S\left(s\right).D\left(s\right)$$

The goal is to minimize the weighted sensitivity function, $S\left(s\right)w_{p}\left(s\right)$ [24]. The main aim of current work is to find a value for K(s) that reduces the magnitude of ${\left\|SG_{d}\right\|}_{\infty }$ and stabilizes the system. This can be achieved by ensuring that the following stipulation is satisfied.

$$\left|S.D\left(j\omega \right)\right|<\left|\frac{1}{w_{p}\left(j\omega \right)}\right|,\left|S\right|<\left|\frac{1}{w_{p}\left(j\omega \right)}\right|,\left|S.K\right|<\left|\frac{1}{w_{u}\left(j\omega \right)}\right|,\text{and}\;\left|S.K.D\left(j\omega \right)\right|<\left|\frac{1}{w_{u}\left(j\omega \right)}\right|\;\forall \omega$$

Or,

(17) $$\left\{\begin{array}{c} \begin{array}{c} \begin{array}{c} {\left\|S.D.w_{p}\left(j\omega \right)\right\|}_{\infty }<1\\ {\left\|S.w_{p}\left(j\omega \right)\right\|}_{\infty }<1 \end{array}\\ {\left\|S.K.w_{u}\left(j\omega \right)\right\|}_{\infty }<1 \end{array}\\ {\left\|S.K.D.w_{u}\left(j\omega \right)\right\|}_{\infty }<1 \end{array}\right\}$$

Based on Fig. 4, $z_{1},E$ and $z_{2}$ may be expressed as:

(18) $$z_{1}=w_{p}{\hat{V}}_{R}-Dw_{p}\hat{F}-Gw_{p}u$$

(19) $$E={\hat{V}}_{R}-D\hat{F}-Gu$$

(20) $$z_{2}=w_{u}u$$

**Fig. 5:**
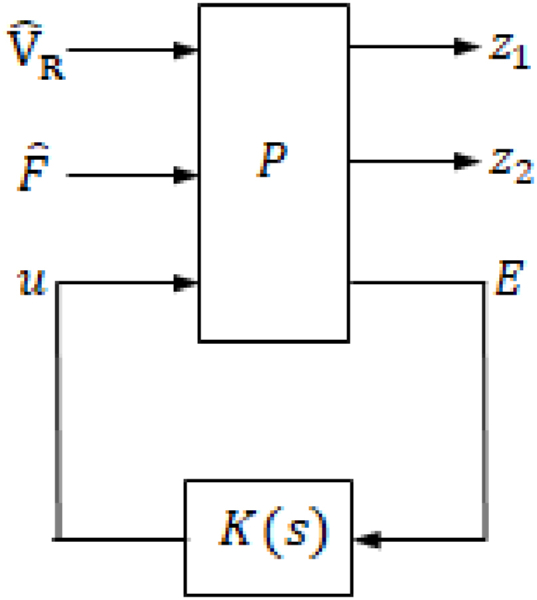
Generalized plant, P.

From Eqs. (18), (19), (20), the generalized plant (P) of the system presented in Fig. 4 is shown in Fig. (5) and is given in Eq. (21).

For analysis purposes, the generalized plant matrix can be partitioned as shown below,

(21) $$P=\left[\begin{array}{cc} P_{11} & P_{12}\\ P_{21} & P_{22} \end{array}\right]$$

where

$$p_{11}=\left[\begin{array}{ccc} 0 & 0 & 0\\ -Gw_{p} & w_{p} & -Dw_{p}\\ 0 & 0 & 0 \end{array}\right],p_{12}=\left[\begin{array}{c} w_{u}\\ -Gw_{p}\\ w_{p} \end{array}\right],p_{21}=\left[\begin{array}{ccc} ‐G & I & ‐D \end{array}\right],p_{22}=\left[-G\right]$$

Using MATLAB®, the transfer function of the $H_{\infty }$ controller, K(s), is determined and is given as.

(22) $$K\left(s\right)=\frac{26.26\;s^{2}+30.62\;s+421.6}{\;s^{3}+44.42\;s^{2}+333.5\;s+1.006}$$

**Fig. 6:**
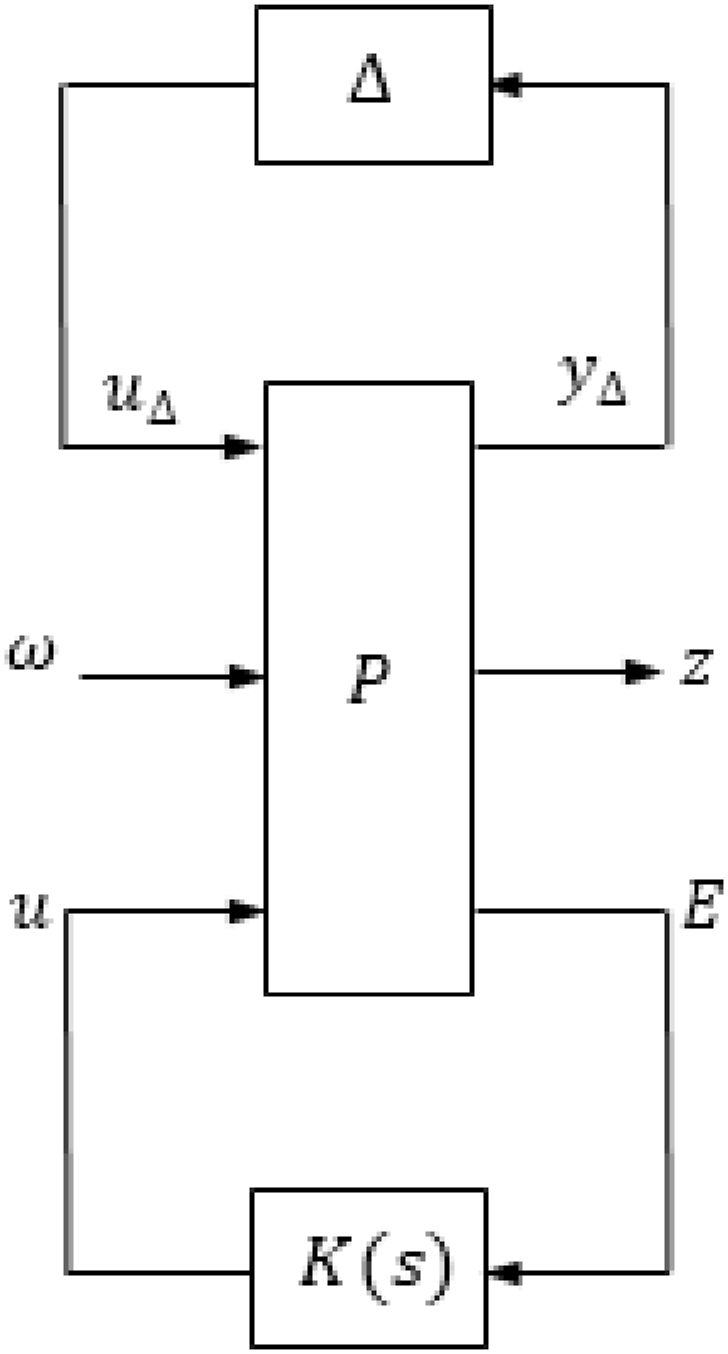
The control configuration.

**Fig. 7:**
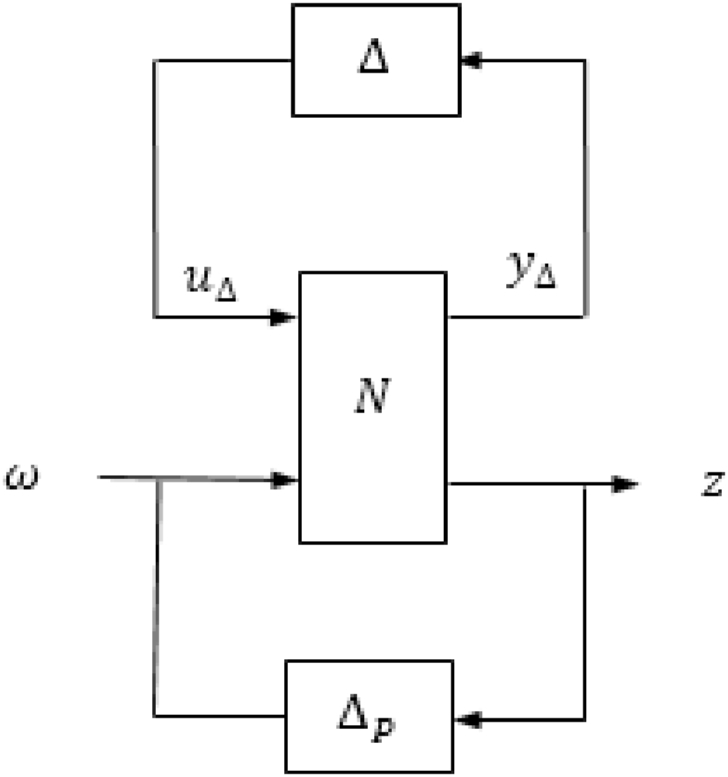
The structure of N−Δ.

The controller given in Equation (22) meets the conditions illustrated in Equation (15) as demonstrated in Fig. 6, Fig. 7.

To investigate the robustness of the system, a set of perturbed plants are generated. These perturbed plants are obtained by introducing a 5% variation in the values of the pump speed, viscous damping coefficient, and actuator efficiency. By using this set of perturbed plants, the relative error, referred to as the multiplicative uncertainty $l_{1}$, can be computed. The multiplicative uncertainty $l_{1}$ is defined as the difference between the frequency responses of the perturbed plant and the nominal plant divided by the frequency response of the nominal plant. Both the multiplicative uncertainty $l_{1}$ and its rational weight $w_{1}$ are expressed in Equation (23) and Equation (24) respectively.

(23) $$l_{I}\left(\omega \right)=\max_{G_{pert}\in \Pi }\left|\frac{G_{pert}\left(j\omega \right)-G_{0}\left(j\omega \right)}{G_{0}\left(j\omega \right)}\right|$$

(24) $$w_{I}\left(j\omega \right)\geq l_{I}\left(\omega \right),\forall \omega$$

In Equations (23), (24), $G_{\text{pert}}\left(j\omega \right)$ represents the perturbed plant and $G_{0}\left(j\omega \right)$ represents the nominal plant.

The structured matrix, which is taken into account when designing the controller to address both uncertainty and performance, is represented by Equation (25).

(25) $$\hat{\Delta }=\left[\begin{array}{cc} \hat{\Delta } & 0\\ 0 & \Delta_{P} \end{array}\right]$$

where Δ is the model uncertainty and $\Delta_{p}$ is the performance uncertainty. Fig. 6 shows the structured matrix with its inputs and outputs. It can be seen that the input and output of the model uncertainty, Δ, are, $y_{\Delta }$, and, $u_{\Delta }$, respectively. In addition, the inputs of the performance uncertainty, $\Delta_{p}$, $\left(z_{1\;}and\;z_{2}\right)$ and the outputs are $\left({\hat{V}}_{R}\;and\;\hat{F}\right)$. Furthermore, the input of the controller, K, is, E, and the output is, u. The matrix of the nominal system, N, is defined in Eq. (26) and is shown in Fig. 7. A lower linear fractional transformation (LFT) relates N to the generalized plant and the controller [24].

(26) $$N=P_{11}+P_{12}K{\left(I-P_{22}\right)}^{-1}P_{21}$$

Based in Fig. 4, the inputs of N are $\left(u_{\Delta },{\hat{V}}_{R},and\;\hat{F}\right)$ and the outputs are $\left(y_{\Delta },z_{1},and\;z_{2}\right)$ which may be expressed as in Eq. (28).

(28) $$\left[\begin{array}{c} y_{\Delta }\\ z_{1}\\ z_{2} \end{array}\right]=\left[N\right]\left[\begin{array}{c} u_{\Delta }\\ \hat{R}\\ \hat{F} \end{array}\right]$$

For investigating the robustness of the system, the nominal matrix N is divided into four partitions $\left(N_{11},N_{12},N_{21},and\;N_{22}\right)$.

## 6 The criteria of stability and performance

**Fig. 8:**
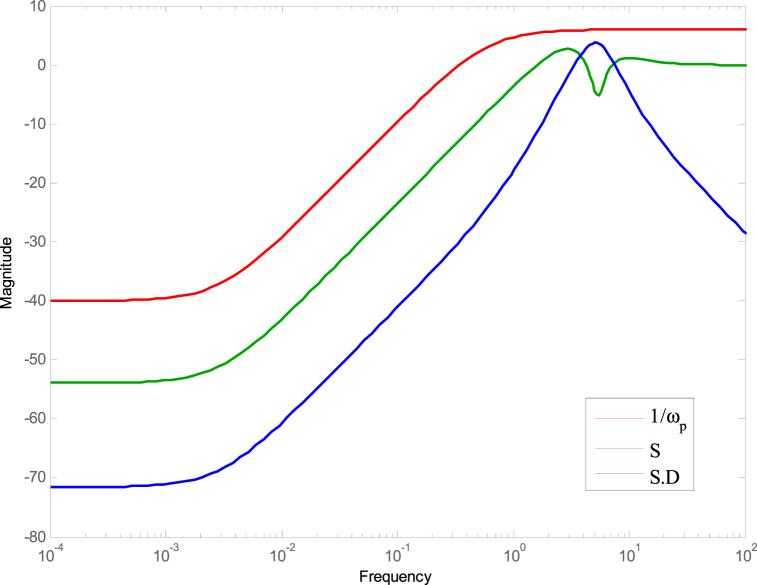
First and second conditions of Eq. (17).

**Fig. 9:**
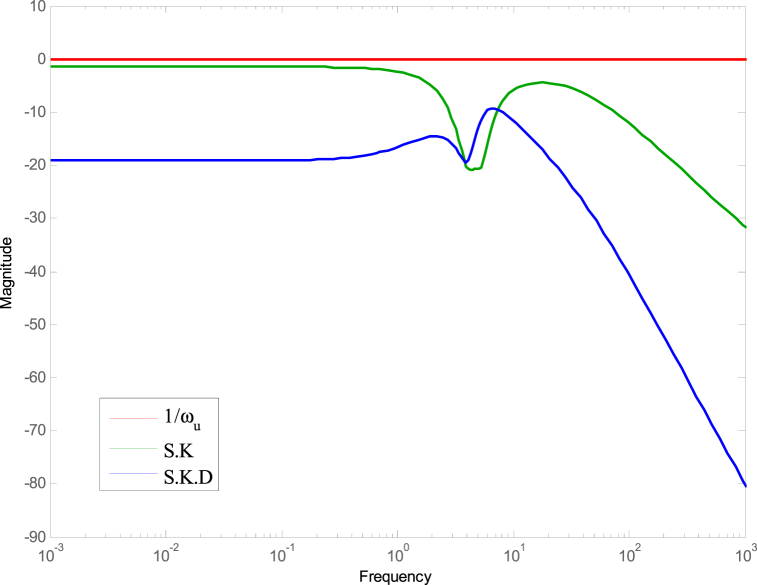
Third and fourth conditions of Eq. (17).

Fig. 8, Fig. 9 demonstrate that the conditions given in Eq. (17) are met. The stability and performance of both the nominal and perturbed systems are analyzed using the nominal matrix N. The criteria of the nominal performance, NP, robust stability, RS, and robust performance, RP, are defined in Eq. (29) [24].

(29) $$\begin{array}{c} NP\Leftrightarrow {\left\|N_{22}\right\|}_{\infty }\;<1\\ RS\Leftrightarrow {\left\|N_{11}\right\|}_{\infty }\;<1\\ RP\Leftrightarrow \mu \left(N,\hat{\Delta }\right)\;<1 \end{array}\}$$

## 7 Results and discussion

**Fig. 10:**
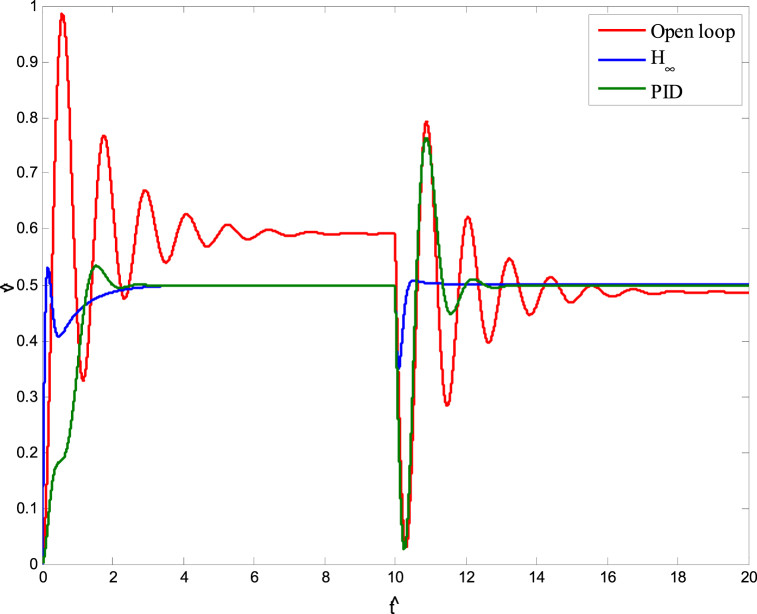
Time response of the dimensionless cylinder velocity.

**Fig. 11:**
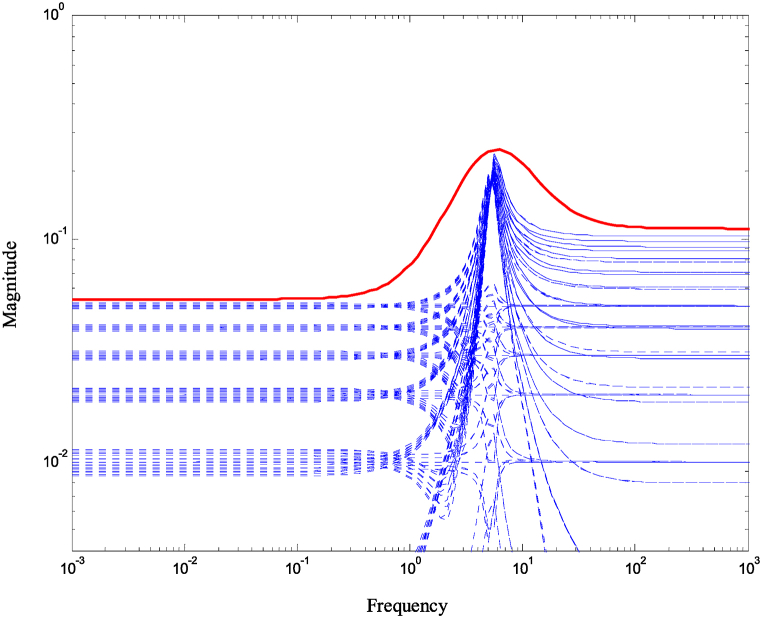
The multiplicative error bound.

**Fig. 12:**
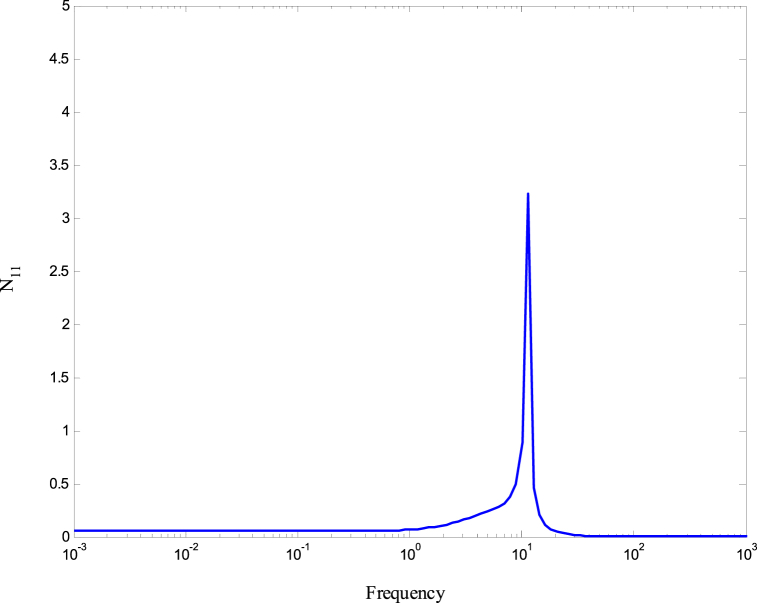
The robust stability bound of the system with PID controller.

**Fig. 13:**
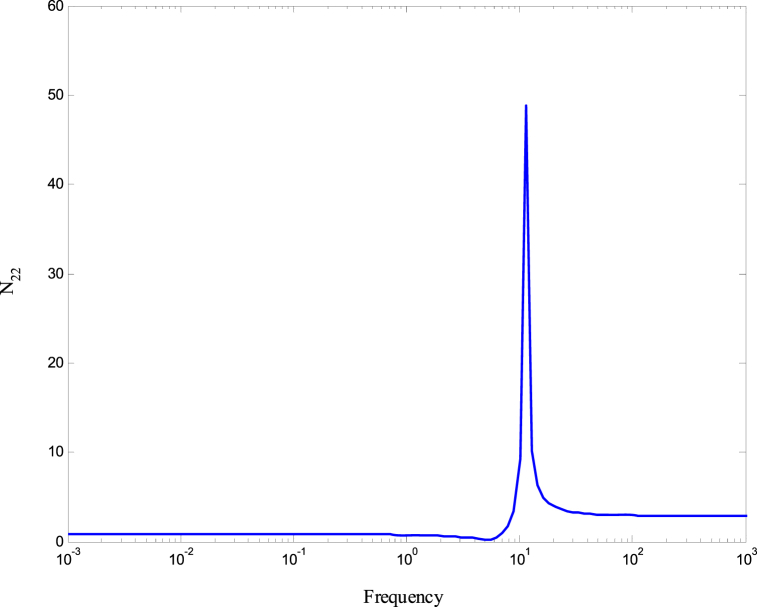
The nominal performance bound of the system with PID controller.

**Fig. 14:**
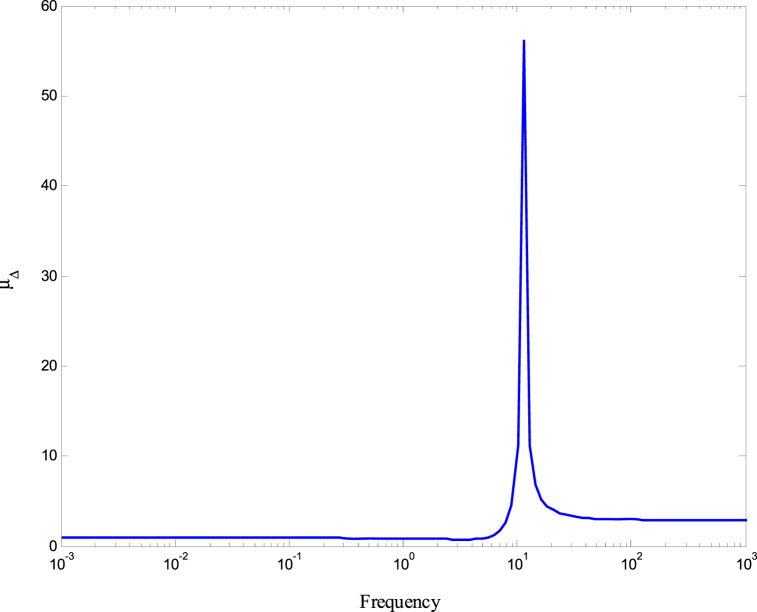
The robust performance bound of the system with PID controller.

**Fig. 15:**
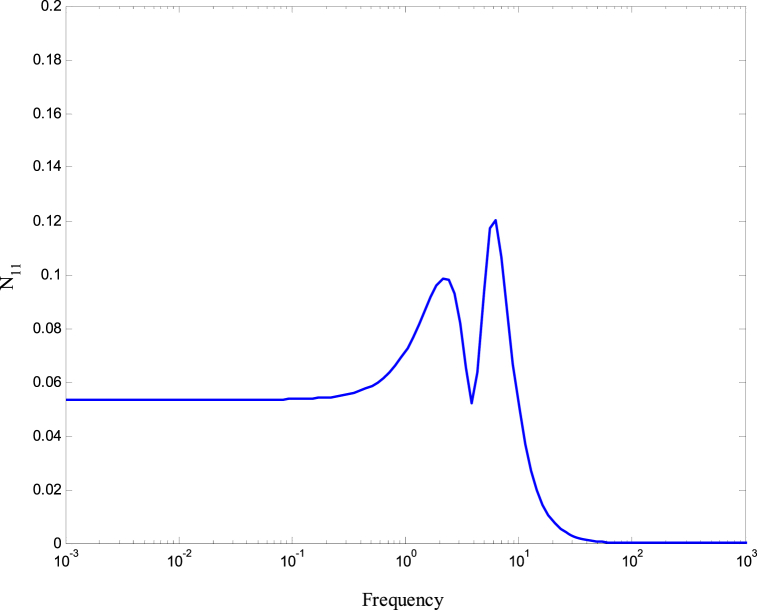
The robust stability bound of the system with H-infinity controller.

**Fig. 16:**
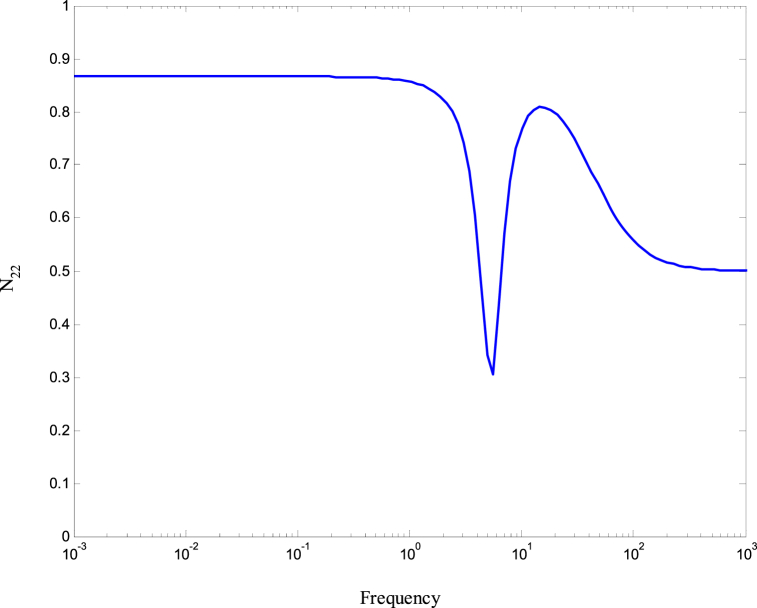
The nominal performance bound of the system with H-infinity controller.

**Fig. 17:**
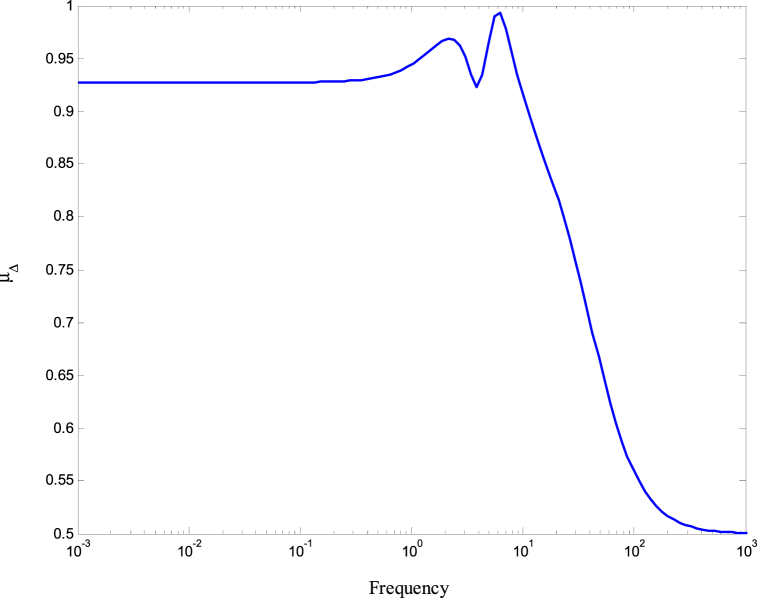
The nominal performance bound of the system with H-infinity controller.

A model was created for a hydrostatic transmission that is used for velocity control purposes. The system's response was analyzed in terms of stability and performance for three scenarios: the open-loop system, the closed-loop system with a PID controller, and the closed-loop system with an H-infinity controller. The robustness of the system was also examined for both the PID and H controllers. In the analysis, a reference velocity of 0.5 was used, and a disturbance with a magnitude of 0.8 was introduced into the system after 10 time constants for both the open-loop and closed-loop configurations. To determine the PID controller gains, the Matlab/Simulink auto-tuning feature was employed, resulting in $\left(K_{p}=2.471,K_{i}=3.66,and\;K_{d}=0.396\right)$ as the selected gains for the PID controller. Fig. 10 shows the time responses of the cylinder velocity for the open-loop and the closed-loop with both controllers. The time response analysis demonstrates that the system remains stable for real-world applications. However, the performance of the open-loop system was found to be inadequate, characterized by excessive overshoot, oscillations, lengthy settling time, and poor disturbance rejection. To enhance the system's performance, a PID controller was designed. The implementation of the PID controller significantly improved the system's response to the reference input, allowing for better tracking. Furthermore, the system's response to the disturbance input was also enhanced, resulting in reduced settling time and improved steady-state error performance. Observing Fig. 10, it is evident that the system response still exhibits a significant overshoot. To further enhance the system's performance, an $H_{\infty }$ controller was designed. Notably, the $H_{\infty }$ controller yielded a remarkable improvement in the system's response to disturbances while maintaining a response to the reference input that closely resembled that of the PID controller. Fig. 11 displays the uncertainty bounds that limit the maximum error, providing insight into the system's robustness. Conversely, Fig. 12, Fig. 13, Fig. 14 depict the requirements for robust stability, nominal performance, and robust performance with the PID controller. It is apparent that none of these requirements were satisfied. However, when the $H_{\infty }$ controller was employed, as shown in Fig. 15, Fig. 16, Fig. 17, all stability and performance requirements were met. This highlights another advantage of utilizing the $H_{\infty }$ controller, as it consistently satisfies the necessary stability and performance criteria.

## 8 Conclusions

The main conclusions of the present work are:

1. The open-loop system is always stable for real world applications.
2. The steady state error is zero with both PID and $H_{\infty }$ controllers.
3. Using PID controllers improves the system input tracking but does not reduce the overshoot and settling time associated with the system response to the disturbance force.
4. The system's requirements for robustness are not met by the PID controller.
5. Using $H_{\infty }$ controller eliminates the overshoot of the system response to the disturbance force and greatly reduces the settling time while keeping the other performance parameters similar to those with the PID controller.
6. $H_{\infty }$ Controller satisfies all robustness requirements of the system.
7. Future works may include experimentally validating the proposed control strategies in addition to considering other control strategies to achieve a give a more intuitive comparison.

## Funding

This research did not receive any specific grant from any funding agency in the public, commercial, or not-for-profit sectors.

## Data and code availability

Has data associated with this study been deposited into a publicly available repository?

No. The data that support the findings of this study are available on request from the corresponding author.

## Ethical statement

Compliance With Ethical Standards.

## Author agreement statement

The authors whose names are listed immediately below declare that this manuscript is original, has not been published before and is not currently being considered for publication elsewhere. We confirm that the manuscript has been read and approved by all named authors and that there are no other persons who satisfied the criteria for authorship but are not listed. We further confirm that the order of authors listed in the manuscript has been approved by all of us. We understand that the Corresponding Author is the sole contact for the Editorial process. He/she is responsible for communicating.

## CRediT authorship contribution statement

**Jabbar H. Mohmmed:** Conceptualization, Writing – original draft. **Ahmed K. Hassan:** Software, Validation. **Muslim Ali:** Conceptualization, Writing – review & editing. **Samer A. Kokz:** Investigation, Resources. **Muhanad Hamed Mosa:** Software, Validation. **A.K. Kareem:** Investigation, Resources. **Ahmed A. Zainulabdeen:** Supervision. **Z.C. Feng:** Supervision.

## Declaration of competing interest

The authors declare that they have no known competing financial interests or personal relationships that could have appeared to influence the work reported in this paper.
